# Low Power Multi-Hop Networking Analysis in Intelligent Environments

**DOI:** 10.3390/s17051153

**Published:** 2017-05-19

**Authors:** Josu Etxaniz, Gerardo Aranguren

**Affiliations:** Electronic Technology Department, University of the Basque Country, Bilbao 48013, Spain; gerardo.aranguren@ehu.es

**Keywords:** ambient intelligence, ACL, Bluetooth, delay, empirical model, intelligent environment, latency, multi-hop, scatternet

## Abstract

Intelligent systems are driven by the latest technological advances in many different areas such as sensing, embedded systems, wireless communications or context recognition. This paper focuses on some of those areas. Concretely, the paper deals with wireless communications issues in embedded systems. More precisely, the paper combines the multi-hop networking with Bluetooth technology and a quality of service (QoS) metric, the latency. Bluetooth is a radio license-free worldwide communication standard that makes low power multi-hop wireless networking available. It establishes piconets (point-to-point and point-to-multipoint links) and scatternets (multi-hop networks). As a result, many Bluetooth nodes can be interconnected to set up ambient intelligent networks. Then, this paper presents the results of the investigation on multi-hop latency with park and sniff Bluetooth low power modes conducted over the hardware test bench previously implemented. In addition, the empirical models to estimate the latency of multi-hop communications over Bluetooth Asynchronous Connectionless Links (ACL) in park and sniff mode are given. The designers of devices and networks for intelligent systems will benefit from the estimation of the latency in Bluetooth multi-hop communications that the models provide.

## 1. Introduction

Intelligent systems are usually included in environments where not only sensing and recognition capabilities are required, but also the collection, processing, changing and sharing of the available context information. Such environments represent a new information technology paradigm and they are also called intelligent environments or ambient intelligence. They consist of the integration of information technologies into everyday objects in order to improve the quality of life. Intelligent systems are a multidisciplinary approach [1], as they require the convergence of many areas of Computer Science in order to fulfill their purpose.

Many devices are usually added to intelligent environments. Specifically, such environments include electronic systems to interconnect nodes and communicate the measured data. Every intelligent device should be able to transmit and receive the context information that is collected by the sensors. The information is usually processed and/or zipped before its transmission [2,3] because the radio transmission at the node consumes more energy than any other operation at the node [4,5]. However, since in many environments the context information usually changes at a low pace, the continuous sensing of that information is not necessary, so it is possible to make the nodes sleep at regular intervals. In fact, intelligent environments composed of wireless multi-hop networks usually require long-life sensor nodes to be interconnected through a communication network. Hence, it is not only possible but also mandatory to work with devices in low power mode in such intelligent environments.

In order to extend the life of nodes, they can be connected to the mains so that there would be no need to care about the power consumption of the nodes. Moreover, when a battery is added to the node, the node would still be fully operational in case of blackouts. When there is no chance to connect the nodes to the mains, they should have a battery power supply and, besides, they must work in low power mode so that the life-cycle of the node is as long as possible. The inactivity intervals of the gateway and sensor nodes can be scheduled to reduce their power consumption and, hence, the consumption of the whole network. However, the inactivity intervals have a deep influence in the degradation of the time performance of the network. Thus, even if the nodes included in the network might be connected to the mains, it is important to reduce the power bill of the whole network without degrading significantly its performance in terms of response time or latency.

Generally speaking, any application that needs to share the measured data for their collaborative use is considered here. There is a wide range of applications with this feature. Some examples of such wireless networks are intelligent environments (smart cities or homes, augmented reality, or ubiquitous computing systems), or some monitoring networks such as environmental (air or water quality monitoring, and landslide or forest fire detection), health care (at hospitals, homes for the Elderly or by means of wearable devices), industrial (structural health monitoring, or machine health monitoring) and area monitoring networks (geo-fencing of pipelines, and enemy intrusion detection). This paper focuses on intelligent environment applications.

In wireless networks, the achievement of the specific Quality of Service (QoS) through the allocation of the limited resources among users is still an open challenge [6]. The allocation of resources [7] can take place according to some performance metrics, such as throughput and fairness [8,9,10,11,12] or according to the traffic types [13,14,15]. However, there are other metrics to evaluate the performance of the network, as the latency in the communication. In fact, the analysis of the time performance of wireless networks is a key point due to many facts [16]. First, there is an increasing use of wireless networks for limited response time applications. Second, the transmission medium is usually unreliable. Finally, there is a significant variability in the communication delay coming from factors as the random delay added whenever the data must be retransmitted and the variable wait since the data are delivered to the Host Controller Interface (HCI) until they are transmitted by radio. As a result, latency is a key metric when defining the QoS of a communication system [17].

There are many wireless communication standards that can be used to implement networks for intelligent environments [18,19]. Bluetooth is a worldwide communication standard without radio emission license costs. It allows for multi-hop networking with low energy consumption. Such features made this wireless technology the chosen one as the communication standard to implement multi-hop networks for intelligent systems. Even though there are differences among the releases of the Bluetooth standard, the chosen release, i.e., the 2.0 one, is still on the cutting edge of the technology in terms of multi-hop transmission performance [20].

Then, the goal was set on obtaining the data that lead to the empirical model for the latency in the wireless communications. Focusing on that purpose, a test bench based on commercially available off-the-shelf (COTS) products was developed. Many prototype nodes were developed with some Bluetooth modules. Such nodes can be integrated into electronic systems for intelligent environments so that they can interconnect via wireless links. Furthermore, it was built the middleware to manage the performance of the node at the HCI level of the radio module. Thanks to the middleware, the nodes were configured using high-level commands. So, the performance of Bluetooth gateways inside multi-hop networks was analyzed in the tests by measuring the end-to-end latency QoS metric. Not only the tests were repeated a lot of times, but also the data analyzed statistically to limit the errors made in examining a finite set of samples.

This paper analyzes the end-to-end latency of the network in order to evaluate the time performance of the multi-hop communication network for intelligent systems. Bluetooth provides several low power modes for the radio transceptor of the nodes. They have been considered two representative Bluetooth low power modes: *sniff*, which keeps the biggest activity and connectivity while nodes are in low power mode; and *park*, which loses the most connectivity when nodes are parked.

To the best of the authors’ knowledge, there is no empirical analysis of the latency in Bluetooth multi-hop communications with low powered modes. This paper introduces and discusses the empirical models for the upper limit of the transmission latency from end-to-end in a multi-hop wireless network when working with Asynchronous Connectionless Links (ACL) in both, park and sniff low power modes. The models will benefit the designers of intelligent systems and wireless networks providing them with the estimation of the latency that every hop in the communication adds.

The paper is organized as follows: Section 2 introduces a case study of intelligent systems networking, as well as the Bluetooth standard multi-hop networking capability and the way it is applied to such systems. Next, Section 3 gives the features of the test bench developed. In addition, Section 4 explains the details of the methodology of the tests. The results of the tests are discussed in the Section 5, as well as some models are proposed for the latency in the case study of multi-hop wireless communications networking. After that, Section 6 discusses the suitability of the low power modes under test for real world applications. Finally, Section 7 summarizes the conclusions and points out the potential future work.

## 2. Intelligent Systems at Home

The latest technological advances impact areas as different as vision algorithms, context recognition, content creation tools, conversational interfaces, ambient database systems, embedded systems, wearable electronics, sensing, ubiquitous communication technologies, or wireless communications are driving intelligent systems [21]. Any environment that includes intelligent systems should be anticipatory, adaptive, or embedded [22]. In addition, Cook et al., considered the features of being transparent, responsive, sensitive, and ubiquitous [18]. Domestic appliances (e.g., lighting, vision, or sound appliances), or electronic devices for personal healthcare that are responsive to the presence of people are frequently contained in this paradigm to collaborate with one another [23].

### 2.1. Case Study: Networking of Intelligent Systems

**Figure 1:**
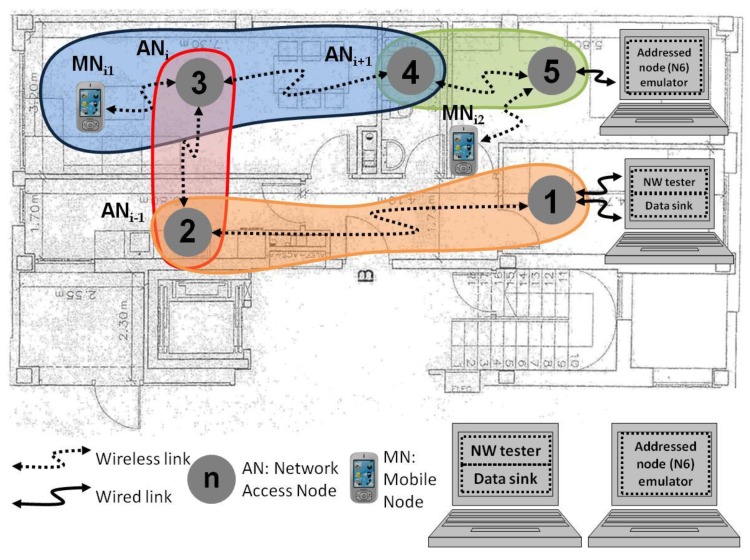
Example of multi-hop network deployment for intelligent systems (distances expressed in meters).

This paper considers the multi-hop network deployment practical scenario for an intelligent environment represented in Figure 1. Even though the diagram represents a network deployment in a home, such a deployment could also take place in assisted living homes, foster homes, hostels, student residences, hotels, or any other analogous buildings. As shown in Figure 1, some wireless access nodes (AN_i_) and mobile nodes (MN_ij_) are included in the network. The MN_ij_, as the AN_i_, can also include some sensors depending on the application of the network. The AN_i_ in Figure 1 are deployed all through the home to provide wireless network connectivity to the MN_ij_. Many types of sensor are included in systems for intelligent environments. They are usually attached to both access and mobile nodes with wireless connectivity. The network in Figure 1 can also include some environmental sensors in the rooms, as for instance, temperature sensors for the centralized air-conditioner system or fire alarms. The nodes would centralize the measured data into a data sink (DS). The alarms and control commands that might be necessary after the data processing would be generated in the DS. Furthermore, several functionalities in the computer connected to the first and the last nodes of the network eased the monitoring process. For instance, the test commands were generated in the network tester (NW tester) and the resultant data were aggregated in the DS.

There are several reasons to deploy a wireless network of access nodes instead of considering the wired solution. On the one hand, the deployment costs of a wireless network are lower than the costs of the wired approach. On the other hand, the installation of the network access nodes is complex, and might be risky, so a wireless network is arranged whenever the budget restrictions, convenience, or other issues make this approach the most suitable one.

Generally speaking, the devices in intelligent environments are interconnected and they form a communication network. Hence, a communication standard must be found to provide connectivity to such devices. Bluetooth [24] is a standard that satisfies these requirements.

Then, in the case shown in Figure 1, every MN_ij_ connects to the most appropriate AN_i_ periodically, once the programmed inactivity period is finished. The requirements in latency depend on the specific application and the type of sensor. As an example, temperature or humidity sensors are usually included in applications with longer monitoring intervals than presence sensors. In case the wireless communication standard offers many low power working modes, as it happens in the case of Bluetooth, it is necessary to choose wisely the low power mode for the best performance of the system.

### 2.2. Bluetooth Multi-Hop Networking

The Bluetooth networking capability is based on *piconets*, i.e., the smallest network that can be formed with this communication technology. Whenever two or more Bluetooth devices share a radio channel, they form such centralized networks called *piconets*. A central node (master), which controls the communications, and up to seven active nodes (slaves) are included in *piconets*. Figure 1 shows some *piconets*. For instance, the one colored in dark blue, where the AN_i_ is the master, and the AN_i+1_ and the MN_i1_ are the slaves.

Moreover, the standard includes the notion of a more complex network, i.e., the *scatternet*, but it does not define the way to form it. Such network not only helps to extend the coverage of wireless devices, but also increases the amount of active nodes in a limited area. Any Bluetooth device can play the role of slave in many *piconets* as well as be the master in at most one *piconet*. When some nodes operate in two or more *piconets*, they are called gateways and a multi-hop network is formed, i.e., a *scatternet*.

There are two types of gateways in Bluetooth, according to the roles they play in the piconets: master-slave and slave-slave. The master-slave gateway offers less delay from end to end for traffic between two adjacent piconets (inter-piconet traffic) [25]. Since the research is focused on inter-piconet traffic delivery through scatternets, the nodes in the test bench were configured as master-slave gateways, i.e., as masters of the piconet formed with the next node, and as slaves with the previous node (according to the numbers of the nodes in Figure 1).

The gateways in Figure 1 are connected in linear topology and they relay the data gathered from both the sensors connected to the AN_i_ in the rooms and the sensors connected to the MN_ij_, which are carried by the monitored users. The first sensors can be those to measure the temperature, detect the presence of people, or even presence of smoke; and the second ones to measure the vital signs or user’s body temperature.

Then, every halfway node in the wireless test bench, i.e., every gateway, participates in two piconets given that it has an open link as a master and another link as a slave. The communications in Bluetooth follow time division multiplex (TDM) scheme defined by the master node. At the baseband level, each slot lasts 625 μs, so the slot rate is 1.6 kHz. Thus, there are inactivity intervals for the nodes inside the piconet. When two nodes establish a link, they negotiate the moments of activity in the piconet and the intervals in which each slave will be transmitting and receiving data. The master node of every piconet in the tests polls slave nodes every 25 ms, as it is set by default. So, nodes can take advantage of the activity breaks in the piconet, where they are slaves, to attend to the piconet where they play the master role, guaranteeing that they can attend to both of the links. Not only the slave nodes but also the master nodes can abandon the piconet while there is no activity and start playing the slave role in the other piconet. But if a node perceives the absence of the other node in the piconet, the node closes the link and the link must be re-established later if it needs to transfer data to the other node. Hence, the latency of the multi-hop communication will increase in such situations.

The standard defines the Basic Data Rate that allows up to 723.2 kbps asymmetric rate with 1 bit per symbol transmission under Gaussian Frequency Shift Keying (GFSK) modulation. In addition, the standard defines the sniff and park low power modes to take advantage of the inactivity intervals. Both modes are analyzed in this paper.

On the one hand, when any node operates in sniff mode, the inactivity intervals (*T_sniff_*) or periods of absence in the piconet are configured such that the slave node agrees with the master to periodically listen to its transmissions. The sniff low power mode is configured at the HCI level and is defined by some parameters: *T_sniff_*, *N_sniff_attempt_* and *N_sniff_timeout_*. A device in sniff mode listens to incoming data periodically with an interval of *T_sniff_*, a value between the *Sniff_Max_Interval* and *Sniff_Min_Interval* parameters, and with the duration specified by the other two parameters. *N_sniff_attempt_* defines the amount of receive timeslots the device will listen to, and *N_sniff_timeout_* describes how many receive timeslots it will listen to after the latest received packet.

On the other hand, when the nodes operate in park mode, some beacons are listened by the slave node every *Beacon_interval*. Meanwhile, the node can be low powered or dedicated to other tasks out of the radio tasks in that piconet. Then, the inactivity interval in park mode (T_park_) was defined by the value for the *Beacon_interval* between the *Beacon_Max_Interval* and *Beacon_Min_Interval*.

### 2.3. Bluetooth Applied to Intelligent Systems

Intelligent systems developers usually make good use of the large amount of technological breakthroughs in the areas of, for instance, wireless sensor systems, wearable electronics, ubiquitous communication, ambient database systems, conversational interfaces, content creation tools, and vision algorithms [21]. As a consequence, an intelligent environment typically is embedded, ubiquitous, anticipatory, responsive, sensitive, adaptive, and transparent [18,22]. The requirements for an environment to be called intelligent are fulfilled by Bluetooth standard [24]. This technology has been included in the data transportation network of many proposals for intelligent environment systems [26,27,28]. Actually, Bluetooth protocols have significant potential to support deterministic behavior, i.e., real-time, asynchronous communication [29].

There are many issues that give relevance to the analysis of the performance of wireless networks. To begin with, there is an increase in the use of wireless networks for constrained response time applications. Secondly, the transmission medium is usually considered as inaccurate. And finally, there is a random increase in latency due to both, the retransmissions that the medium inaccuracy leads to, and the unpredictable delay between the delivery of data to the host interface and radio transmission. This increase make the latency show a non-trivial variability [16].

On the other hand, as a result of the efforts in research on Bluetooth, an extensive literature is available on many aspects of Bluetooth networking. Next, the state-of-the-art of Bluetooth networking performance analysis is summarized. In fact, the performance is assessed by means of QoS metrics. There are two types of metrics: traffic dependent or traffic independent [30]. The firsts require the specification of a defining source, packets destination and traffic profile. Besides, packet flows are considered. As a consequence, the evaluation of traffic dependent metrics is difficult, and traffic independent performance measures are frequently used. The most common of these last metrics are the bit error rate (BER), the throughput and the latency. For instance, the first two of the traffic independent metrics, i.e., the BER and throughput, were analyzed under noise and interference conditions [31] in scatternets. Moreover, a mathematical model to analyze the performance of Bluetooth data links was provided [32], in terms of many metrics, latency included. But none of the results of these researches were validated in hardware test benches so that the model proposals get close to the real world. In the same way, some other analytical approaches to the performance of piconets were validated with simulation engines [33,34,35].

Furthermore, there are several proposals of theoretical models to define the communications inside piconets. For instance, the latency in asynchronous communications in piconets was studied [36], as well as analyzed when particularized for connections using serial port profile [37]. Besides, it was shown that the latency in the communications in a piconet follows a step outline with the length of the data packets as a result of the data segmentation [38]. All of them were empirically validated in actual piconets.

On the other hand, the concept of file transfer delay (FTD) was introduced in a piconet, which included the time delay of the packet, and the delays due to encapsulation/de-encapsulation, signal propagation and retransmission. Then, an empirical model was proposed to foresee the FTD in a piconet without theoretical support [39].

On the whole, scatternets have barely been analyzed in terms of latency. The latency in a three node scatternet was determined [40]. It included a slave-slave gateway. Even though they pointed out that the communication between a master and a slave node are not symmetric and involve different latency values, it is a fact that a real world scenario should include more than one hop in the wireless network.

All in all, real world applications designers in intelligent environments need models of the main metrics of wireless networks, so that they can determine the application range of a specific wireless standard. In fact, low power modes with a defined period of inactivity are almost mandatory in intelligent environments. Since theoretical calculations or simulation engines cannot foresee all the real-world issues, empirical models are required to analyze the viability of specific wireless applications [41]. Just a few empirical models on latency have been already obtained in Bluetooth multi-hop networks [20,42]. As Bluetooth does not support synchronous links in scatternets, what is needed are empirical models for the latency in scatternets with ACL links in low power modes.

## 3. Test Bench

**Figure 2:**
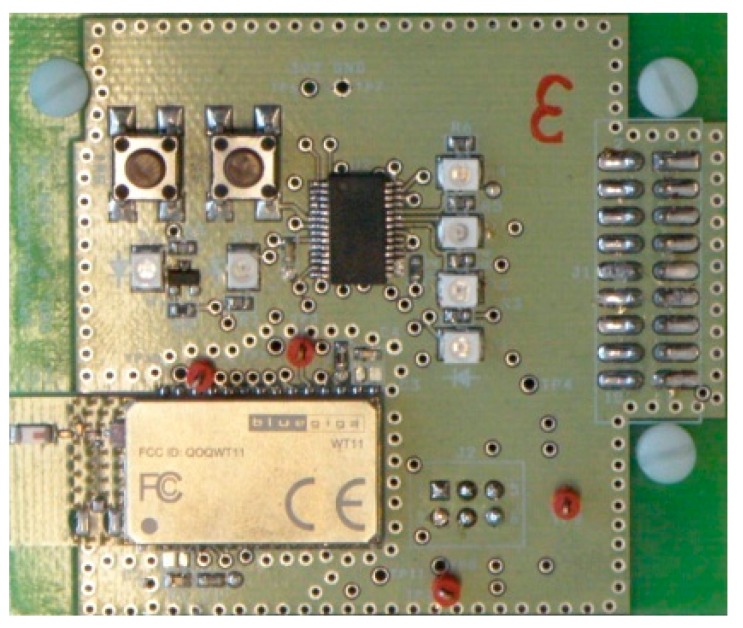
Photography of one of the implemented Bluetooth nodes.

After implementing some proprietary nodes with Bluetooth connectivity, the test bench was deployed. It was focused on studying the latency on multi-hop communications and included as many gateways as possible in a scatternet. The test bench consisted of a multi-hop wireless network of proprietary nodes based on Bluetooth technology. Figure 2 shows one of the implemented nodes for this research. The nodes included the WT-11 chipset of Bluegiga [43], as well as some general-purpose input and output components and test pins. The chipset included a firmware developed by Cambridge Silicon Radio [44], named BlueCore4 [45], that eases the low power modes defined in the Bluetooth specifications and analyzed in this manuscript, i.e., park and sniff. BlueCore4 includes the HCI Stack (version 19.2) to allow the development of proprietary middleware tailored to the specifications of the application. The proprietary middleware was designed using BlueLab [46], the software tool to develop embedded software given by the manufacturer of the chipset [44]. BlueLab eases the programming of the nodes at the HCI level, the lowest level that any proprietary middleware can access within the Bluetooth protocol stack. Moreover, the middleware included the processing tasks, which were limited to the most basic operations to restrict their influence in the latency. They integrated processes to initialize the communications, present the operation status and route data to other nodes. The data reached the node and they were stored in the input buffer until the processor attended the interruption flag. Similarly, the routines below the HCI level included in the standard define the wait since the data are delivered to the HCI level buffer until they are transmitted by radio. Since the processing tasks in the node were limited, such delay can be assigned to the regular operation of the communication standard.

**Figure 3:**
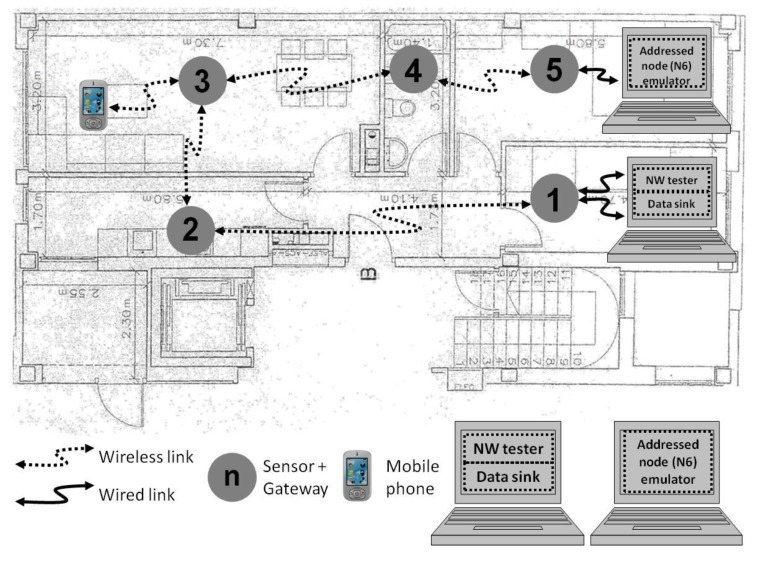
Example of multi-hop network deployment for an intelligent environment inside a home (distances expressed in meters).

On the other hand, there is a wide variety of intelligent environment examples. They are wherever the ambiance can recognize people, personalize to individual preferences or even act upon their behalf [23,47]. The research in this paper takes into account the deployments of multi-hop networks based on Bluetooth standard inside buildings, as it is the home deployment depicted in Figure 3. Here, the network access nodes are depicted as grey circles. Some sensors, as smoke or temperature ones, would be attached to the nodes, and the network could be accessed by wireless devices, as mobile phones. Besides, even though the mains are usually at hand in such indoor deployments, a battery is included in each node to overcome any failure in the mains that might happen.

In the test bench, each pair of nodes built a piconet, and the interconnection of piconets formed a multi-hop network with linear topology. It can be said that the linear topology is one of the simplest topologies. However, it is the most suitable topology to study the performance of the desired amount of Bluetooth gateways with the least amount of nodes. In the network deployment, every halfway node performs as gateway and relays the data gathered from other sensor nodes (not depicted) to the DS. The DS keeps all the information available in the system and shares with all the devices through the multi-hop network. On the other hand, there is a network administrator that accesses the data in two ways. They can gain admission to the network through a mobile phone with Bluetooth connectivity or through a computer connected to the node labeled as number one.

## 4. Methodology

In order to come near the intelligent home environment network deployment, the tests presented in this paper were carried out in the test bench deployed in the laboratory of the research group. The nodes were spread in the laboratory and the corridor nearby. Firstly, it was checked that the medium range distances considered in the following tests had no significant influence in the latency of the communication. After that, it was applied the methodology to analyze the performance of master-slave gateways in scatternets described in [48].

The proprietary middleware in the nodes of the test bed considers the Basic Data Rate, as well as some specific values to define the parameters for the low power modes under test. For instance, the sniff low power mode is defined by the amount of desired slots for the *Sniff_Max_Interval*, two slot less for the *Sniff_Min_Interval*, one slot for the *N_sniff_attempt_* and eight slots for *N_sniff_timeout_* parameters. In addition, the park low power mode is defined by the amount of desired slots for the *Beacon_Max_Interval*, and two slots less for the *Beacon_Min_Interval* parameters.

The scheme of interconnection of nodes in the test bench is shown in Figure 3 for the case of four hops in the communication. It included a computer connected to the end nodes, i.e., the first and last ones of the network, to ease the monitoring process. The NW tester and the addressed node emulator (ANE) functionalities were built-in the computer. All the functionalities, i.e., NW tester, DS and ANE, were synchronized because they shared the computer. Thus, the time that the data packets took hopping from slave to master nodes could be measured, in addition to the time taken from master to slave nodes.

So, the tests were carried out as explained next. First, the NW tester functionality sends *ping* type data packets to the last node of the wireless test network, i.e., node N6 in Figure 3. The halfway nodes receive the packets, process and resend them towards the addressed node. The ANE functionality in the computer receives the *ping* packet, logs the reception moment and sends the answer packet while logging the transmission moment. The answer consists of a *pingback* type data packet. Since the communication follows the TDM scheme, both the *ping* and *pingback* packets, fit in a time slot defined in the Bluetooth standard (625 µs). So, the *pingback* packet flows along the network, going from slaves to masters of the piconets and in the end, it reaches the computer connected to the first node of the network. The DS functionality included in the computer logs the reception moment of the *pingback* packet. The latencies for both the *ping* packets (master-to-slave communication) and the *pingback* packets (slave-to-master) are obtained as the differences between the logs in both ends of the network. Therefore, each *ping* packet went hopping from node to node until it reached the destination node. Similarly, the *pingback* packets went back to the source node following the same path. The following *ping* packets were sent right after the *pingback* packet reached the source node. This way, the *ping* packets were randomly sent, as the sending interval was defined by the latency of the network, which turned to be not constant and with some deviation in the tests. The tests were repeated numerous times (a total amount above 12,000 samples were taken in park mode and almost 11,000 samples in sniff mode). The 95% percentile of the latency distribution for each one of the situations considered was chosen to be analyzed because it provides us with the upper limit of the latency. This statistic datum helps the network engineers to design the management of limited response time systems or intelligent environments with the latency limit estimation of multi-hop communications over Bluetooth Asynchronous Connectionless Links (ACL) in park and sniff mode.

Finally, the effect of the delay by retransmissions due to interference was extracted from the sets of samples after analyzing them and not considering the outliers of the series. Such retransmissions are usually a result of the influence of a series of external factors, as the presence of other equipment in the same frequency band or the other processes run by the operating system in the computer. On the other hand, the resolution of the instrumentation used in the tests was about tens of milliseconds and it was enhanced by repeating the tests hundredths of times for each one of the situations.

## 5. Discussion of the Results

Given that this paper analyzes the latency in multi-hop networks with Bluetooth standard, both the time that the *ping* type data packets take to go from the first to the last node, as well as the time that the *pingback* data packets take to go the other way round were measured. Moreover, two low power link policies were considered; *park* and *sniff*, and several periods of inactivity were tested, namely 100, 500, 1000 and 2000 ms. For all the tests, the amount of nodes in the network, and hence the amount of hops, was changed. An average of 320 samples per situation under test was considered in park mode, and 270 samples per situation in sniff mode.

### 5.1. Multi-Hop Communications with Bluetooth Park Low Power Mode

**Figure 4:**
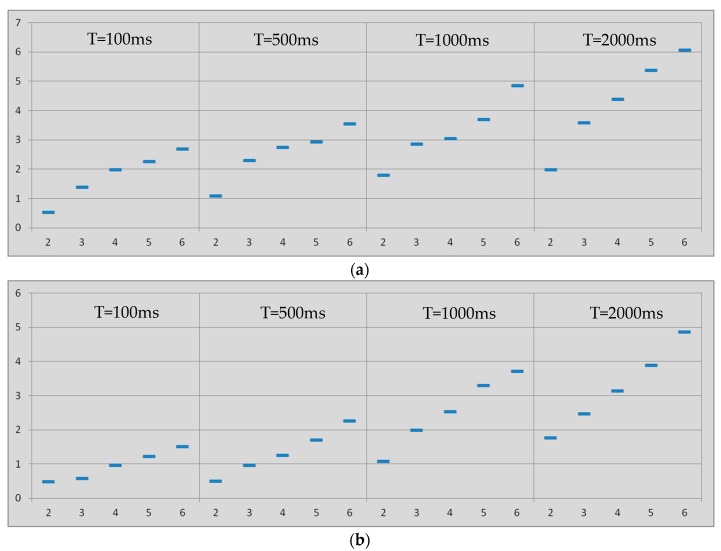
Latency (vertical, seconds) vs. amount of hops (horizontal). Links always in *park* mode. Inactivity intervals (from left to the right): 100, 500, 1000 and 2000 ms. (**a**) *master-to-slave* communication; (**b**) *slave-to-master* communication.

As a result of the tests with nodes in park mode, several figures are available to analyze the latency in Bluetooth multi-hop networks. In all cases, latency is expressed in seconds in the ordinates axis. Figure 4a summarizes the 95% percentile of the results of the latency tests vs. amount of hops for the master-to-slave communication when links in the network are always in the park mode.

The results when the communication is from slave to master are compiled in Figure 4b. In these two figures the four cases of inactivity intervals (T) were considered. The percentile of the measured latencies depicted in Figure 4 shows a monotonically increase when the amount of hops is increased.

The latency results when operating in park mode show higher values for the case of data packets going from master to slave (Figure 4a) when compared to data packets going from slave to master (Figure 4b). The reason behind such unexpected results can be found in the methodology of the tests. The slave-to-master data packets (i.e., *pingback* data packets) for the DS flow along the network as soon as they are received in the ANE, and therefore, the pingback data packets find some slave nodes active, i.e., there is no need to unpark those links and hence the latency is not increased.

**Figure 5:**
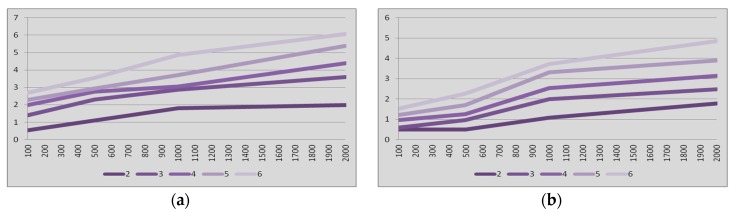
Latency (vertical, seconds) vs. inactivity interval (horizontal, milliseconds). Links always in *park* mode. From two to six hops: (**a**) *master-to-slave* communication; (**b**) *slave-to-master* communication.

On the other hand, Figure 5a shows the dependence of the latency with the inactivity interval in the same case, i.e., master-to-slave communication when links are in park mode. Furthermore, the dependence of the latency with the inactivity interval for the slave-to-master communication when links are in the park mode can be seen in Figure 5b. In these two figures five cases are depicted depending on the amount of hops considered, i.e., from two to six hops.

Figure 5 confirms what Figure 4 points out, i.e., the latency from end-to-end in Bluetooth multi-hop wireless communications when links are always in the park mode exhibits linear dependence with the inactivity period of the nodes. The latency also depends on the amount of hops in the communication. This phenomenon happens not only in the master-to-slave communication but also in the slave-to-master communication.

Figure 4 also indicates that the latency from end-to-end in Bluetooth multi-hop wireless communications when links are always in the park mode exhibits slight quadratic dependence with the amount of hops. This phenomenon is related to the probability of errors in the communication and the subsequent retransmissions, which increases as the communication includes more hops. It happens not only in the master-to-slave communication but also in the slave-to-master communication.

These results are the basis to propose a model for the latency in asynchronous communications along Bluetooth multi-hop networks when master-slave gateway nodes are in park low power mode. The model describes and anticipates the upper limit of the latency in Bluetooth networks with linear topology. It embraces the four inactivity periods considered in the tests, i.e., 100, 500, 1000 and 2000 ms. The communication from master to slave is defined with the same model that defines the communication from slave to master, but the coefficients change, as shown next.

The achievement of the models followed these steps. First, the results of the test were analyzed, and the dependencies of the latency with the amount of hops of the communication and the period of inactivity were extracted. Then, the coefficients of the master equation for the cases under study were defined. Next, linear regression was performed to calculate the coefficients of the equations that define the models. Then, the models were defined. After that, the models were validated with the measurements through a graphical comparison as well as the R^2^ statistical factor and the mean squared error (MSE). R^2^ is used in the context of a statistical model which pretends to predict future results or to test a hypothesis. It determines the quality of the model to replicate the results and the part of the variation of the results that can be explained with the model [49]. On the other hand, the MSE is a measure of the quality of an estimator that measures the average of the squares of the errors or deviations, i.e., the difference between the estimator and what is estimated [50].

Taking into account the dependences shown in Figure 4 and Figure 5, the end-to-end latency when gateways nodes are in the park mode can be defined as in Equation (1), which considers the time contributions described in [48] and includes the delay time of the first hop (*t*_1*park*_), the amount of hops (*N*), the latency contribution per hop (*δ*_1_), the latency addition in each hop (2 *δ_2_*), the inactivity period of the mode (*T_park_*), and the ratio of inactivity period affecting the latency (*β*):

(1) $$\delta_{park}=t_{1park}+\sum_{i=2}^{N}\left(\delta_{1}+\beta \cdot T_{park}+2\cdot \delta_{2}\cdot \left(i-1\right)\right)$$

Then, the values for the coefficients in Equation (1) were calculated through linear regression to define the curves that best fit to the results of the measurements for the park mode. The values are summarized in Table 1 for both the multi-hop communication from master to slave and from slave to master when links are in the park mode. Table 1 also includes the results of the validation with R^2^ statistical datum and the results for the MSE.

As a summary, the upper limit of the latency in asynchronous links of Bluetooth multi-hop networks can be defined with just one equation for each one of the directions, i.e., master-to-slave and slave-to-master communication in park low power mode. Furthermore, the models were validated with the R^2^ statistical parameter and the MSE.

### 5.2. Multi-Hop Communications with Bluetooth Sniff Low Power Mode

**Figure 6:**
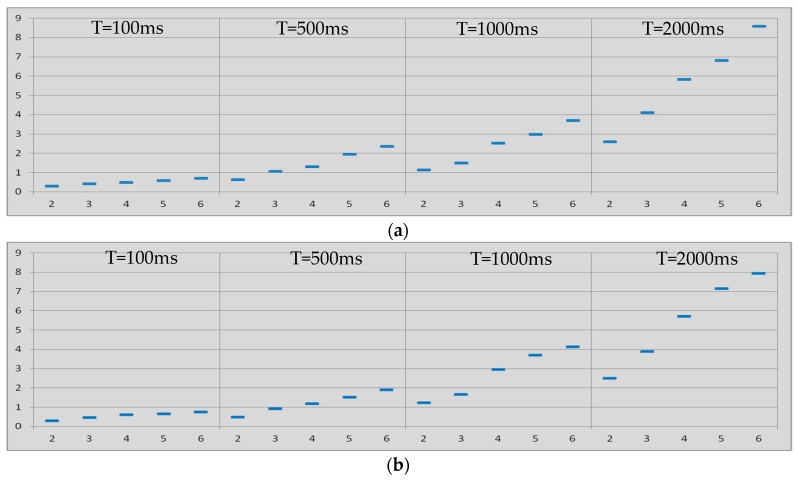
Latency (vertical, seconds) vs. amount of hops (horizontal). Links always in *sniff* mode. Inactivity intervals: 100, 500, 1000 and 2000 ms. (**a**) *master-to-slave* communication; (**b**) *slave-to-master* communication.

On the other hand, several figures are available to analyze the latency in Bluetooth multi-hop networks with nodes in sniff mode. Figure 6a summarizes the 95% percentile results of the latency tests vs. amount of hops for the master-to-slave communication when links in the network are always in the sniff mode. The results when the communication is from slave to master are compiled in Figure 6b. As in the park mode, in these two figures four cases of inactivity intervals were considered, namely 100, 500, 1000 and 2000 ms.

Figure 6 shows that the latency in communication from master to slave when links are in the sniff mode is very close to the latency in the communication from slave to master. There is no major difference between these two latencies. The point is that the sniff mode can be exited by either the master or the slave sending a specific request command so that the requested device replies with the according acceptance command. If the master requests an exit from sniff mode it shall enter sniff transition mode and then send an *LMP_unsniff_req* command. On the one hand, when the slave receives the *LMP_unsniff_req* it shall exit from sniff mode and reply with an *LMP_accepted* command. On the other hand, when the master receives the *LMP_accepted* command it shall exit from sniff transition mode and enter active mode.

**Figure 7:**
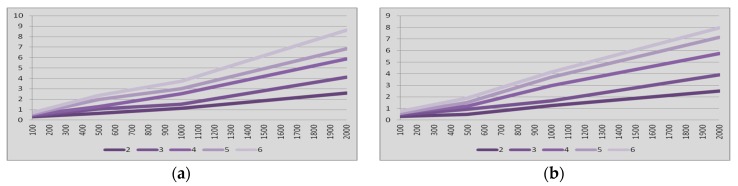
Latency (vertical, seconds) vs. inactivity interval (horizontal, milliseconds). Links always in *sniff* mode. From two to six hops. (**a**) *master-to-slave* communication; (**b**) *slave-to-master* communication.

Figure 7a shows the dependence of the latency with the inactivity interval in the same case, i.e., master-to-slave communication when links are in sniff mode. The dependence of the latency with the inactivity interval for the slave-to-master communication when links are in the sniff mode can be seen in Figure 7b. As in the park mode, in these two figures five cases are depicted depending on the amount of hops considered, i.e., from two to six hops.

Figure 7 confirms what Figure 6 points out, i.e., the latency from end-to-end in Bluetooth multi-hop wireless communications when links are always in the sniff mode exhibits linear dependence with the inactivity period of the nodes. The latency also depends on the amount of hops in the communication. This phenomenon happens not only in the master-to-slave communication but also in the slave-to-master communication.

In addition, Figure 7 points out that the latency from end-to-end in Bluetooth multi-hop wireless communications when links are always in the sniff mode exhibits linear dependence with the amount of hops. It can be understood that there is no significant additional delay in the multi-hop communication due to errors or retransmissions. This phenomenon happens not only in the master-to-slave communication but also in the slave-to-master communication.

These results are the basis to propose a model for the latency in asynchronous communications along Bluetooth multi-hop networks when master-slave gateway nodes are in sniff low power mode. The model describes and anticipates the upper limit of the latency in Bluetooth networks with linear topology. It embraces the four inactivity periods considered in the tests, i.e., 100, 500, 1000 and 2000 ms. The communication from master to slave is defined with the same model that defines the communication from slave to master, but the coefficients change, as shown next.

The achievement of the models followed the same steps followed for the case of park mode. Taking into account the dependences shown in Figure 6 and Figure 7, the end-to-end latency when gateways nodes are in the sniff mode can be defined as in Equation (2), which considers the time contributions described in [48] and includes the delay time of the first hop (*t*_1*sniff*_), the amount of hops (*N*), the latency contribution per hop (*δ*_a_), the inactivity period of the mode (*T_sniff_*), and the ratio of inactivity period affecting the latency (*α*):

(2) $$\delta_{sniff}=t_{1sniff}+\sum_{i=2}^{N}\left(\delta_{a}+\alpha \cdot T_{sniff}\right)$$

Then, the values for the coefficients in Equation (2) were calculated through linear regression to define the curves that best fit to the results of the measurements summarized in Figure 6 for the sniff mode. The values are summarized in Table 2 for both the multi-hop communication from master to slave and from slave to master when links are in the sniff mode. Table 2 also includes the results of the validation with R^2^ statistical datum and the results for the MSE.

As a summary, the upper limit of the latency in asynchronous links of Bluetooth multi-hop networks can be defined with just one equation for each one of the directions, i.e., master-to-slave and slave-to-master communication in sniff low power mode. Furthermore, the models were validated with the R^2^ statistical parameter and the MSE.

## 6. Selection of the Most Suitable Low Power Mode

This paper introduces research on the performance of the park and sniff low power modes defined in the Bluetooth standard specification [24]. More precisely, it was the time performance the one under test in the experiments carried out during the research. Both low power modes, sniff and park, provide a useful way to reduce the power bill of a pair of connected Bluetooth devices, a master and a slave node. They are eligible when no active participation in the piconet is needed, but the slave device wants to remain synchronized.

The results from the experiments summarized and discussed in Section 5 demonstrate that there is no only one low power mode that provides the best performance when operating in Bluetooth multi-hop networks. The choice depends on the following factors discussed in this section. To begin with, the key points of sniff and park modes are summarized.

On the one hand, while in sniff mode, the master and slave devices exchange ACL packets only on specific timeslots. Therefore, the slave needs to be ready only for these timeslots. In fact, the absences scheduled throughout extended periods of time allow the slave to save power by turning off its radio. As a result, the battery operating time is reduced and the power bill decreased. Alternatively, sniff mode may also be used by a device that participates in multiple piconets, also known as scatternet, when it needs to perform some action in another piconet.

On the other hand, while in park mode, even though a slave does not actively participate in a piconet, it remains synchronized to the master. Hence, the slave must wake up at regular intervals to resynchronize and listen for packets. When slaves are in park mode all traffic is stopped, ACL and Synchronous Connection Oriented (SCO). Actually, only small data transmission is enabled for maintaining the synchronization. The master supports a beacon channel to allow the broadcast traffic, the piconet synchronization, and the unpark negotiation. Then, the data traffic is only enabled right after finishing the unpark procedure.

Park mode offers some advantages to the management of nodes in a piconet. On the one hand, park mode allows more than seven slaves to participate in a single piconet, and on the other hand, it allows, as sniff mode also does, to reduce the power consumption of the nodes as they are only active at regular intervals.

However, park mode shows inherent problems in terms of QoS [51]. For instance, the traffic scheduling to more than the seven active slave nodes requires simultaneous indirect scheduling for park/unpark procedures, making it unpredictable and leading to data packet losses. Moreover, the unpark procedure is not deterministic and it can last as much time as the supervision timeout, which is set to 20 s per default. Therefore, since no latency guarantees on the unpark procedure are provided, instead of parking a node, it could be better to disconnect it and reconnect it just when the node needs to be addressed once more. But another solution can be found in the specification [24]. It consists of operating in sniff mode with the master node transmitting *null* packets instead of *poll* packets, so that the slave does not have to respond to them and, therefore, it can save power. When the network operates in such way, the slave node has to respond only when the master requires an update of the supervision timeout. So, since there are no known interoperability problems with sniff mode (the specification for sniff mode is very simple compared to the one for park mode), and the system is less complex (which reduces its cost), such alternative solution provides better performance.

**Figure 8:**
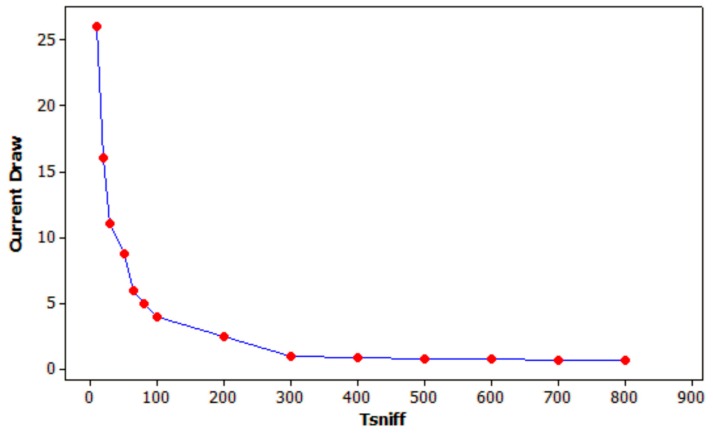
Current consumption vs. inactivity interval in sniff mode, *T_sniff_*.

All in all, the focus of this paper is set on the time performance of the multi-hop networks and the subsequent power consumption reduction of the low power modes under test, sniff and park. It is out of the scope the way to handle more than seven slaves in a single piconet. Then, the power consumption in both modes is almost the same [51]. In fact, the sniff mode uses a little less power because of two facts: the sniff mode does not require regular Link Management Protocol (LMP) negotiations, and the beacon channel signaling in park mode requires more bandwidth. Figure 8 shows the reduction of the current consumption when the inactivity interval in sniff mode, *T_sniff_*, increases [52]. It shows a general trend where small values of *T_sniff_* provide minimal power savings, but larger values show diminishing returns. As *T_sniff_* becomes longer, the slave must expand its uncertainty window to allow for clock drift, so it uses more power. Then, in the situation depicted in Figure 8, the best choice to set *T_sniff_* that provides the best latency-power consumption trade-off is about 100 ms, because there is a rapid power increase for shorter intervals, and the little additional power savings for longer intervals do not justify the decrease in the responsiveness of the system.

Then, the main factors that must be taken into account to select the most suitable Bluetooth low power mode deployments in intelligent environments are the power consumption of the network and the latency QoS metric. Both sniff and park low power modes provide almost the same power saving when the inactivity interval increases. So the key difference between these modes must be found in terms of QoS, i.e., latency. Negri and Zanetti looked for the best mode power-wise for a link under particular traffic conditions [53]. Sniff mode was found to be particularly indicated in piconets when data duration is small compared to data period while park mode results effective when data duration is longer. Cano et al., showed that sniff offers low power consumption and low transmission delay inside a piconet [54]. Furthermore, in multi-hop communications along a scatternet, the experiments in this paper demonstrate that sniff provides smaller latency than park when the inactivity interval is about 1 s or less. Such inactivity interval optimizes not only the responsiveness of the system for most of the applications that can be found in the real world, but also the latency-power consumption trade-off of the system. The only issue to take into account when operating in sniff mode is the amount of slaves that would simultaneously need to participate in each one of the piconets of the multi-hop network.

Generally speaking, sniff low power mode is the best choice when compared to park low power mode for multi-hop communications with Bluetooth standard with burst pattern data traffic, i.e., short data packets. Such data packets usually are in many applications for the Internet of Things (IoT) [55]. Actually, the latest version of the Bluetooth specification is focused on applications for IoT and it does not support the park low power mode. Then, each application must evaluate their specific needs and determine a suitable value for the sniff interval through experiment running. Moreover, the value set in all of the sniff mode parameters depends on the use case and the role assignments for the nodes involved. Therefore, the best way to set these values is to carry out interoperability tests and try different combinations of values. Then, it will be easy to find out which set of parameters works best when considering the trade-off between power consumption and latency.

## 7. Conclusions

The electronic systems for intelligent environment applications are usually embedded, adaptive, as well as sensitive and responsive to the presence of living beings in such a way that the technology integrates into environments smoothly. Given that Bluetooth standard initially focused on replacing the cables of electronic equipment, Bluetooth provides intelligent environment systems with low latency and low power wireless connectivity. It is a wireless technology extended all over the world that saves the license costs of radio emission. Bluetooth provides the establishment of low power multi-hop networks. An important QoS metric of any communication network is the latency in the transmission of data. In fact, this metric is a major problem for multi-hop networks.

Even though there is a vast literature about Bluetooth, it features a gap relative to the analysis of the latency in multi-hop communications. Latency depends on several factors. So, in this paper the dependences of latency with the major factors, namely the hop count, the power mode of the nodes (sniff and park) and the period of inactivity are studied.

Furthermore, the results of the tests derived in the set of equations to calculate the upper limits of the latency in communications over ACL links in scatternets when links are in *park* or *sniff* modes. The equations are the first approximation in terms of latency to model the performance of low power modes in Bluetooth multi-hop wireless networks. They set the foundation for future approximations where the processing stage of the nodes or the topology of the network would be more complex. Therefore, the set of equations benefits the designers of devices for intelligent environments and wireless networks. It gives them the estimation of the upper limits of the latency that each hop of the network would add when setting, for instance, an intelligent environment with a wireless network of access points.

None of the low power modes under test offered always the lowest latency results in the tests. So the modes were characterized to make a good choice. As the systems for intelligent environments can be divided into long and short monitoring period systems, the most suitable low power mode depending on the specifications of the application is set in this paper.

Finally, the future work includes the use of devices of the very last release of Bluetooth standard to provide a model that extends the topology, and the network application scenario.

## Figures and Tables

**Table 1:** Values of the coefficients in Equation (1) for master-to-slave and slave-to-master multi-hop communication with nodes in park mode.

Coefficient	Master-to-Slave	Slave-to-Master
*t*_1*park*_ (ms)	990	410
*δ*_1_ (ms)	250	145
*β* (%)	40	38
*δ*_2_ (ms)	10	8
R^2^	0.9527	0.9396
MSE	0.0914	0.0895

**Table 2:** Values of the coefficients in Equation (2) for master-to-slave and slave-to-master multi-hop communication with nodes in sniff mode.

Coefficient	Master-to-Slave	Slave-to-Master
*t*_1*sniff*_ (ms)	230	320
*δ*_a_ (ms)	14	15
*α* (%)	82	78
R^2^	0.9773	0.9801
MSE	0.1161	0.1088

## Abbreviations

The following abbreviations are used in this manuscript:

QoS	Quality of Service
ACL	Asynchronous Connectionless Links
HCI	Host Controller Interface
COTS	Commercially available Off-The-Shelf
DS	Data Sink
AN_i_	Access Node
MN_ij_	Mobile Node
NW	Network
TDM	Time Division Multiplex
GFSK	Gaussian Frequency Shift Keying
BER	Bit Error Rate
FTD	File Transfer Delay
ANE	Addressed Node Emulator
MSE	Mean Squared Error
SCO	Synchronous Connection Oriented
LMP	Link Management Protocol
IoT	Internet of Things
